# Magnetic Resonance of Pulmonary Nodules in Oncological Patients: Are We Ready to Replace Chest CT in Detecting Extrathoracic Cancer?

**DOI:** 10.3390/curroncol32120710

**Published:** 2025-12-16

**Authors:** Ronak Kundalia, Jessica Gemmell, Ian Griffin, Amanda Acevedo, Joice Prodigios, Sandro Bertani, Alysson Roncally Silva Carvalho, Rosana Souza Rodrigues, Hiren J. Mehta, Bruno Hochhegger

**Affiliations:** 1 College of Medicine, University of Florida, Gainesville, FL 32610, USA; jessicashapiro@ufl.edu (J.G.); ian.griffin@ufl.edu (I.G.); amanda.acevedo@ufl.edu (A.A.); 2 Department of Radiology, University of Florida, Gainesville, FL 32610, USA; prodigiosj@ufl.edu (J.P.); bhochhegger@ufl.edu (B.H.); 3 Department of Radiology, Santa Casa de Misericordia de Porto Alegre, Porto Alegre 90020-090, Brazil; sandro@mediscan.com.br; 4 IDOR Institute, Rio de Janeiro 22281-100, Brazil; alysson.carvalho@idor.org (A.R.S.C.); rosana.rodrigues@idor.org (R.S.R.); 5 Division of Pulmonary, Critical Care and Sleep Medicine, University of Florida, Gainesville, FL 32610, USA; hiren.mehta@medicine.ufl.edu

**Keywords:** pulmonary nodule, MRI, cancer

## Abstract

Objective: This study aims to assess the accuracy of pulmonary nodule detection via MRI compared to the gold standard, CT, in patients with extrathoracic cancer. Materials and Methods: MRI of the chest was performed on oncological patients for staging extrathoracic cancer and subsequently compared to their CT. Only the largest nodule was considered in patients with multiple nodules. Nodule size and malignancy were recorded for each modality, as well as the presence of interstitial lung disease (ILD), adenopathy, cardiomegaly, pleural effusion, pericardial effusion, and vertebral fracture. All cases were read by two thoracic radiologists and any discrepancies were resolved by discussion. Results: A total of 154 patients with nodules were identified from 241 participants (mean age: 59 years [18–95]). Average nodule diameter was 11.5 mm (range: 3.9–29.1 mm; SD: 8.1 mm). MRI detected all nodules greater than 5 mm. Malignancy was detected in 37 nodules. The sensitivity, specificity, and accuracy values of MRI for all nodules were 93.51%, 100%, and 95.85%, respectively. For ground-glass nodules (*n* = 11), the values were 43.6%, 100%, and 65.0%, respectively. When compared to CT, long-axis diameter measured by MRI was underestimated by 9 ± 2.3% (*p* < 0.001). There was a strong correlation between measurements of CT and MRI (κ = 0.70–1.00). Furthermore, MRI accurately detected the presence of adenopathy (97.1%), cardiomegaly (99.17%), pleural effusion (98.34%), pericardial effusion (100%), and vertebral fracture (99.6%). Conclusions: These findings suggest that lung MRI accurately detects pulmonary nodules and other thoracic pathologies commonly observed in oncological patients. Lung MRI may serve as a substitute to CT for oncological patients undergoing routine extrathoracic surveillance, thereby decreasing radiation exposure.

## Simple Summary

Patients with extrathoracic cancer require regular thoracic imaging to monitor for pulmonary nodules, which may indicate early metastatic disease. Computed tomography is the current standard, but it exposes patients to repeated doses of ionizing radiation. This study evaluated whether magnetic resonance imaging can accurately detect pulmonary nodules and related thoracic abnormalities in these oncological patients. In more than two hundred individuals undergoing cancer staging, thoracic magnetic resonance imaging was directly compared with computed tomography. Magnetic resonance imaging detected all nodules larger than five millimeters, showed strong agreement with computed tomography for nodule size, and correctly identified all malignant nodules when diffusion-based sequences were used. It also accurately detected lymph adenopathy, cardiomegaly, pleural effusion, pericardial effusion, and vertebral fractures. These findings suggest that magnetic resonance imaging has sufficient diagnostic performance to serve as a safe, effective alternative for routine thoracic surveillance in extrathoracic oncology patients, potentially reducing cumulative radiation exposure while maintaining high diagnostic confidence. Lung MRI accurately detects pulmonary nodules and other thoracic pathologies commonly observed in oncological patients.

## 1. Introduction

Currently, pulmonary nodules are detected in about 1.6 million patients every year and incidentally found in about 30% of CT chest images [1,2,3,4,5]. Surveillance for pulmonary nodules is an essential part of follow-up care for patients with extrathoracic cancers. Malignancies such as breast, colorectal, prostate, and renal cancer frequently metastasize to the lungs [6]. Therefore, the use of imaging to accurately detect pulmonary nodules and metastasis is crucial in improving the survival rates of patients with various types of extrathoracic cancers [3,4,5,6].

MRI has been shown to be an adequate and promising radiation-free alternative to CT in the detection of both solid and subsolid pulmonary nodules larger than 4–5 mm, depending on sequence and scanner field strength [2,3]. The sensitivity for the detection of pulmonary nodules is not only high but also accurately assesses the diameter of nodules [4]. Additionally, MRI has also been found to be an appropriate alternative to CT for patients with cancers originating outside the lung, such as advanced malignant melanoma [4]. Early detection is especially pertinent to these types of oncological patients, as it is estimated that 20–54% of malignant tumors elsewhere in the body lead to pulmonary metastasis [5]. Thus, early detection and diagnosis are of utmost importance to the aim of reducing mortality from this disease. Currently, MRI is widely used for the assessment and staging of many extrathoracic cancers such as breast cancer, prostate cancer, and melanoma. Given recent technological advancements, MRI is capable of accurately detecting pulmonary nodules with near equivalent accuracy to CT in nodules greater than 5 mm [3]. This provides a radiation-free alternative for oncologic patients in the screening and assessment of pulmonary nodules. In other studies, MRI demonstrated a detection rate of 90.2% in nodules > 4 mm, which is comparable to that of CT [2]. Thus, given its ability to accurately detect pulmonary nodules, whole body MRI can be a useful alternative for the detection of cancer metastasis in extrathoracic cancers. Specifically in patients with malignant melanoma, MRI was found to be significantly more specific than CT (83.4% vs. 50.4%, *p* < 0.0001) and only slightly less sensitive (73.4% vs. 78.2%, *p* = 0.0744) in the detection of lesions in patients with malignant melanoma [7]. Provided near equal performance, MRI has demonstrated to be a promising alternative to CT in the assessment of pulmonary nodules.

This study aims to assess the accuracy of pulmonary nodule detection via magnetic resonance imaging (MRI) compared to the gold standard, computed tomography (CT), in patients staging extrathoracic cancer.

## 2. Materials and Methods

The study received approval from the Institutional Review Board at three participating centers. From November 2022 to January 2024, a total of 251 consecutive patients were recruited for this multi-center prospective study. The inclusion criteria for this study were individuals who were over 18 years of age, had extra pulmonary oncological staging with a clinical request of abdominal MRI, and were willing and able to have MRI and participate in the study. The exclusion criteria included contraindications to MR imaging, such as pacemakers, metal implants, and severe claustrophobia, as well as individuals under the age of 18. As per the study protocol, the maximum duration between the CT scan and MRI was 14 days. Ultimately, 241 patients constituted the cohort because there was not enough clinical data (5 patients) or reference imaging studies (5 patients without CT study performed). Therefore, the study group consisted of 241 patients, with 109 being male. The average age of the patients was 60 years, ranging from 18 to 95 years.

### 2.1. Computed Tomography (CT)

Computed tomography was performed utilizing two different CT scanners: a 16-row scanner called Light Speed and a 64-row scanner called Optima CT660, both manufactured by General Electric in Milwaukee, WI, USA. The scan was performed using the following parameters: a collimation of 1.25 mm, a table feed per rotation ranging from 0.938 to 0.984 mm, a tube voltage of 120 kV, and a milliampere-second range of 150 to 300. Images were reconstructed using both lung and mediastinal kernels with iterative reconstruction (ASIR-V, GE Healthcare). The process of reconstructing images was carried out in both axial and coronal orientations with a slice thickness of 3 mm.

### 2.2. Magnetic Resonance Imaging (MRI)

**Figure 1:**
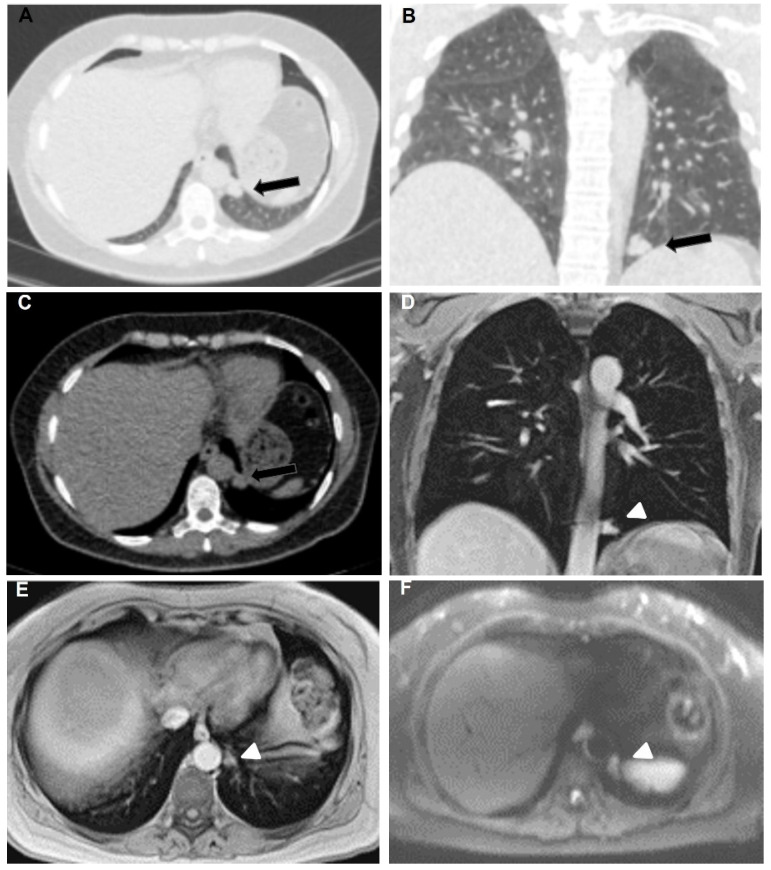
53-year-old female with breast adenocarcinoma. Axial (**A**) and coronal (**B**) lung window and axial (**C**) mediastinal window CT chest show solid, well-defined, lobulated, noncalcified pleural nodular lesion in the left hemithorax (black arrows). Coronal (**D**) and axial (**E**) Gd-enhanced T1-weighted chest MRI images feature the same nodule (arrowheads) as demonstrated in CT. DWI b800 axial chest MRI (**F**) shows diffusion restriction.

**Figure 2:**
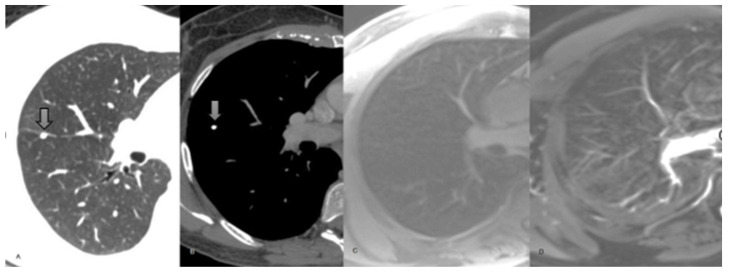
42-year-old female presenting with renal cancer. (**A**) Axial non-enhanced CT chest, lung window. (**B**) Axial T1-weighted non-enhanced chest MRI. Both images show a 5 mm calcified perifissural nodule. (**C**) Axial T1-weighted chest MRI. (**D**) Axial T2- weighted chest MRI. The calcified nodule demonstrated in the chest CT scan is missed in the MRI. The nodule was stable for 6 months and was considered benign.

**Figure 3:**
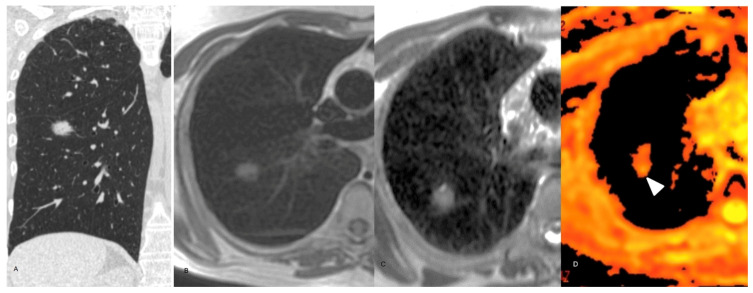
62-year-old man staging colon cancer. (**A**) Coronal reconstruction non-enhanced CT chest, lung window, showing a spiculated 8 mm nodule in the superior segment of the right lower lobe. (**B**) Axial T1-weighted non-enhanced chest MRI demonstrating the same nodule. (**C**) Axial T2-weighted chest MRI. The nodule exhibits high signal in both sequences. (**D**) DWI axial chest MRI showing marked diffusion restriction (arrow head). The final diagnosis was metastatic adenocarcinoma with lepidic growth.

**Figure 4:**
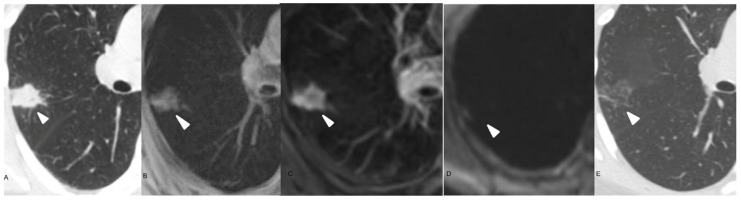
68-year-old male. (**A**) Axial non-enhanced CT chest, lung window, showing a consolidation in the right lung (arrow head). (**B**) Axial T1-weighted non-enhanced chest MRI exhibits a high signal lesion with poorly defined margins (arrow head). (**C**) Axial T2-weighted non-enhanced chest MRI. The lesion has predominantly high signal. (**D**) DWI sequence without remarkable restriction. (**E**) Three-month follow-up chest CT scan demonstrating significant lesion improvement.

The MRI scans were conducted using a 1.5-T machine (Magnetom Avanto, Siemens Medical Solutions, Erlangen, Germany) equipped with three gradients. The gradients had a maximum strength of 40, 40, and 45 mT/m along the x, y, and z axes, respectively, with a slew rate of 200 mT/m/ms. A volumetric interpolated breath-hold examination (VIBE) sequence was chosen for fast T1-weighted MRI and was performed in axial and coronal planes. Imaging parameters for the VIBE sequence were TR/TE, 5.12/2.51 ms; flip angle, 10°; partition thickness, 2 mm with no interslice gap; and matrix size, 256 × 116 with a three-dimensional breath-hold imaging technique (Figure 1 and Figure 2). A T2-weighted fat-saturated BLADE (proprietary name for periodically rotated, overlapping parallel lines with enhanced reconstruction in MR systems from Siemens Healthcare) sequence was also used, with the following imaging parameters: TR/TE, 4670/113 ms; and partition thickness, 5 mm with no interslice gap. DWI was performed using a single-shot echo-planar technique with a slice thickness of 5 mm under spectral attenuated inversion recovery, with respiratory-triggered scanning. The diffusion-weighted imaging (DWI) parameters were TR/TE/flip angle, 3000–4500 ms/65 ms/90°; diffusion gradient encoding in three orthogonal directions; b = 0 and 800 s/mm^2^; field of view, 350 mm; and matrix size, 128 × 128 (Figure 3 and Figure 4). The overall time spent in the chest MRI was approximately 10 min. No patient required sedation.

### 2.3. Image Assessment

The assessment of CT and MR images, together with the measurements of detected lesions, was conducted using commercial workstations, specifically the Leonardo workstation from Siemens Medical Solution in Erlangen, Germany, and the Advantage workstation from GE Healthcare in Milwaukee, WI, USA. Two radiologists, one with 16 years of expertise and the other with 8 years of experience in CT imaging, assessed CT scans after reading MRI datasets. Readers were aware that patients were sent for MRI scans to examine lung nodules. CT and MRI datasets were reviewed in separate reading sessions, and a 4-week washout period was used between modalities to minimize recall bias. During the initial review, each radiologist interpreted the images independently and was blinded to the other modality’s findings and to the other reader’s assessment. The disparities among radiologists, regarding the identification and quantification of abnormalities, were eliminated using a consensus interpretation, conducted independently for CT and MR images. The detection of lung lesions was assessed for all MRI sequences. If there was a discrepancy between MRI sequences, the positive result (the discovered nodule) was selected and the cumulative sensitivity of all MRI sequences was computed. A false negative MRI result was defined as the presence of a nodule (confirmed by CT) that was not identified on specific MR images. The frequency of incorrect positive diagnoses was recorded for each MRI scan. The MR image was used to quantify the maximal diameter of the nodule, ensuring the most accurate delineation of the lesion.

**Figure 5:**
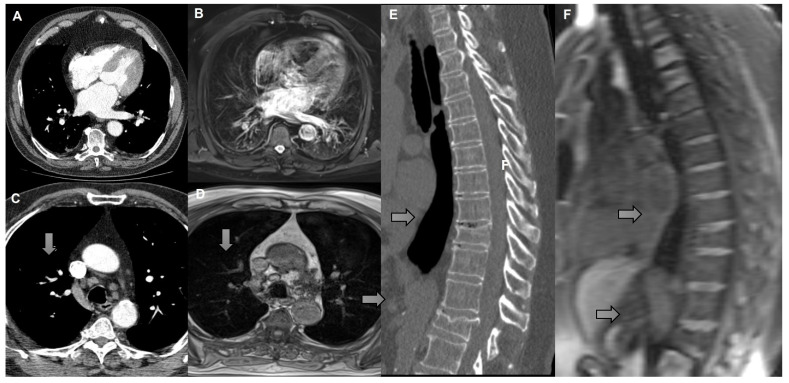
(**A**) Axial CT scan and (**B**) T2 with fat saturation MRI demonstrating cardiomegaly (left atrial enlargement). (**C**) Axial CT scan and (**D**) T1 with VIBE MRI demonstrating right lower paratracheal lymph node measuring 1.1 cm in short axis (arrow). (**E**) Sagittal reconstruction CT scan and (**F**) sagittal reconstruction T1 with VIBE MRI demonstrating vertebral fractures (arrow).

Nodule malignancy was confirmed by histopathology or radiologic progression on follow-up CT/PET-CT with agreement in multidisciplinary meetings. The radiologists also evaluated for the presence or absence of adenopathy, cardiomegaly, pleural effusion, pericardial effusion, vertebral fracture, and aortic (Ao) and pulmonary artery (PA) measurements (Figure 5).

### 2.4. Quantitative Data Analysis

The statistical analysis was conducted using the STATISTICA 10.0 software package developed by StatSoft, Inc., in Tulsa, OK, USA, as well as the MedCalc 9.5.2.0 software package developed by MedCalc Software bvba in Ostend, Belgium. Given the possibility of several pulmonary nodules among participants, a data analysis technique was employed that focused on each individual patient. The presentation of continuous data includes the median and interquartile range (IQR), whereas categorical variables are shown as numbers and percentages, when applicable. The Mann–Whitney U test and Chi-squared test were employed to compare categorical and continuous data, respectively, when deemed appropriate. Spearman’s rank correlation coefficient was utilized to examine the associations between the dimensions of nodules measured using MRI and CT. The detection rate of pulmonary nodules was computed for the entire MRI dataset. Individual studies have been conducted for all nodules, as well as for certain subgroups of lesions, based on the recommendations of the Fleischner organization. These subgroups include nodules measuring smaller than 5 mm, and nodules measuring greater than 5 mm. The diagnostic sensitivity of MRI was determined as the ratio of nodules found by both CT and MRI to the total number of nodules identified in CT scans. A Bland–Altman analysis was conducted to assess the concordance between the CT and MRI measurements of the maximal diameter of the nodule.

## 3. Results

### 3.1. Patient and Nodule Characteristics

In total, 241 patients were included in this study, with an average age of 59 years (18–95) and with 132 females. Nodules were identified in 154 patients (Table 1). No nodules were found in the remaining 91 patients. A total of 88 nodules were found in lower lobes. The average nodule diameter was 11.5 mm (3.9–29.1 mm). In total, 37 nodules were classified as malignant while 107 were benign.

### 3.2. MR Findings

MRI detected all nodules greater than 5 mm. Malignancy was detected in 37 nodules. The sensitivity, specificity, and accuracy values of MRI for all nodules were 93.51%, 100%, and 95.85%, respectively (Table 2). For ground-glass nodules (*n* = 11), the values were 43.6%, 100%, and 65.0%, respectively. MRI accurately detected the presence of adenopathy (95%), cardiomegaly (96.7%), pleural effusion (97.4%), pericardial effusion (100%), and bone lesions (99.3%). DWI was technically adequate in 144 patients. The accuracy of DWI to detect nodule malignancy was 87.66%, detecting all malignant nodules (Table 2).

**Figure 6:**
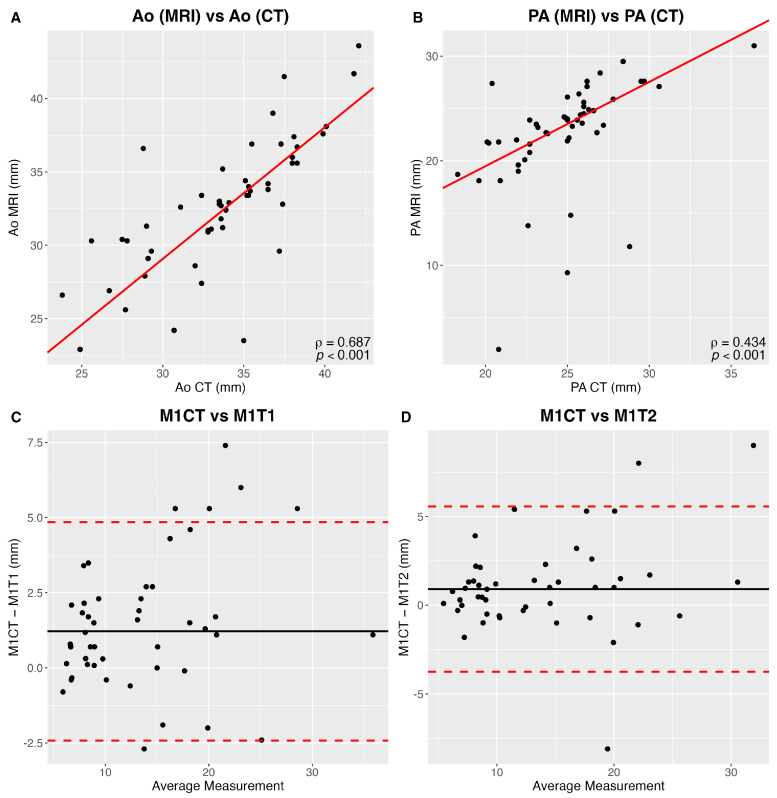
(**A**) Correlation between aortic (Ao) CT and MR measurements. (**B**) Correlation between pulmonary artery (PA) CT and MR measurements. (**C**) Reader 1 (M1) Bland and Altman comparison CT and MR T1-weighted nodule largest axial diameters. (**D**) Reader 1 (M1) Bland and Altman comparison between CT and MR T2-weighted nodule largest axial diameters.

When compared to CT, long-axis diameter measured by MRI was underestimated by 9 ± 2.3% (*p* < 0.001). There was a strong correlation between measurements of CT and MRI (κ = 0.70–1.00) (Figure 6). 

## 4. Discussion

Of the 154 pulmonary nodules detected by CT, the sensitivity, specificity, and accuracy values of MRI for detection of all pulmonary nodules were 93.51%, 100%, and 95.85%, respectively. All 144 nodules that were greater than 5 mm in size were detected by MRI. Diffusion-weighted imaging detected nodule malignancy with a sensitivity of 100% and accuracy of 93.75%. Furthermore, MRI showed considerable accuracy when compared to CT for the detection of adenopathy (97.1%), cardiomegaly (99.17%), pleural effusion (98.34%), pericardial effusion (100%), and vertebral fracture (99.6%). Our study utilized a 1.5 Tesla scanner rather than a 3 Tesla scanner for thoracic imaging. 1.5T scanners provide higher signal-to-noise ratio (SNR) and contrast-to-noise ratio (CNR) for lung parenchyma and small airway visualization, regardless of reconstruction algorithm used, and generally provide superior imaging quality for pulmonary nodule evaluation compared to a 3T scanner [8].

In our study, MRI detected the presence of ground-glass nodules with a sensitivity of 43.6%. This is not necessarily a limitation in the context of pulmonary surveillance for extrathoracic cancers since most pulmonary metastases present as well-defined, solid nodules of variable sizes with/without lymphatic interstitial thickening [9]. A ground-glass nodule in this setting is an atypical metastatic pattern that indicates hemorrhagic metastasis, which is more commonly associated with angiosarcoma or choriocarcinoma [9]. Furthermore, in the setting of primary thoracic ground-glass nodules, Liu et al. demonstrated no significant differences in long-term survival between surveillance and surgery or between stable and increasing sizes, indicating that surveillance for the nodules was appropriate until a solid component emerges [10].

Another important clinical consideration in the follow-up of pulmonary nodules is the accuracy and reproducibility of size measurements. In our study, both T1- and T2-weighted MRI sequences showed strong correlation with CT, with MRI underestimating nodule size by less than 10%. RECIST trials demonstrate that a size discrepancy greater than a 20% increase or 30% decrease between MRI and CT measurements is not considered clinically significant [11]. In addition, prior studies have demonstrated that interscan variability in MRI-based measurements is minimal and does not alter clinical management, supporting the feasibility of MRI for nodule surveillance and screening [12].

Furthermore, underestimation of lesion size on MRI appears to be less relevant when assessing lesion growth on follow-up examinations. In a study of 239 COPD patients with 240 nodules larger than 3 mm, growth of more than 2 mm was observed in 19 nodules and regression of more than 2 mm in 24 nodules after 3 years, with excellent concordance compared to CT (κ = 0.88–1.0) [13]. As current recommendations for LDCT lung cancer screening increasingly emphasize computer-assisted lung nodule volumetry, it is noteworthy that this approach has already been successfully tested in a pilot study using UTE MRI for lung nodule evaluation [14].

Compared to previous studies assessing CT and MRI for the evaluation of pulmonary nodules, our results are similar. In a cohort of 40 patients, Stolzmann found that the detection rates of pulmonary nodules between modalities were similar [5]. Furthermore, they reported a high inter-reader agreement regarding nodule location (k = 0.93–0.98) and size in CT and MRI. Our study found a strong correlation between CT and MRI measurements of pulmonary nodules (k = 0.70–1.00). Another previous study comparing detection of pulmonary nodules between CT and MRI in fifty patients found that the modalities had similar specificity and sensitivity [4]. Similarly to our data, the authors report that MRI sensitivity increased as the diameter of the nodule increased [4]. Also, there was no significant difference between the maximum diameter of pulmonary nodules measured by CT and MRI. The overall sensitivity of MRI was 80% in nodules that ranged from 2 to 28 mm and 100% in nodules with a diameter of 8 mm or greater. Our study reported a sensitivity of 93.51% for MRI. Sommer et al. reports a sensitivity and specificity for MRI to be 48% and 88%, respectively, compared to CT in the detection of nodules in 49 patients. MRI sensitivity was 78% for malignant nodules and 36% for benign nodules [15]. One important cause of this variability is slice thickness: Sommer et al. uses a slice thickness 50% greater than previously published works [15]. Furthermore, a meta-analysis of 10 studies including 1354 patients and 2062 CT-detected pulmonary nodules found that MRI had a sensitivity of 87.7% for detecting nodules 4 mm or larger and 98.5% for nodules that were 8–10 mm [16]. Patients with no pulmonary nodules were reliably classified as negative by MRI compared with CT, with a false-positive rate of 12.4% [16].

Among patients already diagnosed with cancer, data regarding optimal investigation of pulmonary nodules is scarce [17,18]. Usually, the emergence of nodules during treatment or follow-up raises suspicion for metastasis to the lungs. However, during initial investigation, these findings are challenging. Mery et al. evaluated 1104 patients who underwent solitary pulmonary nodule resection, including 288 patients with a history of extrapulmonary malignancies. They found that older age, smoking history, and larger nodule size were associated with malignancy [19]. Given that a previously published expert review demonstrates that nodules that ultimately prove to be neoplastic grow after a mean of 65 days after initial staging, a follow-up interval of 2–3 months is reasonable for small pulmonary nodules without overtly suspicious imaging features [20,21]. This data favors the decrease in significance of small sub-5 mm pulmonary nodules in oncological patients and reinforces the possible use of MRI in this clinical setting.

Pulmonary metastasis is a common finding in extrathoracic malignancies, in which twenty percent of metastatic disease is isolated to the lungs [22]. The most common finding for pulmonary metastasis is pulmonary nodules, in which the most sensitive modality for detection is CT [23]. However, oncological patients routinely undergo regular abdominal MR screening as the current standard of care to adequate staging of extrathoracic cancers. The addition of Chest MR alongside abdominal MR screening in a ‘one-shot’ study can have the same accuracy in detection of pulmonary metastasis and decrease the overall staging time. This would help to reduce repeated exposure to ionizing radiation for at-risk populations, including pregnant patients and children [2]. An inherent limitation to this notion would be the additional scanning time inherent to MR image acquisition. However, a possible solution involves utilizing the 15 min protocol for thoracic MRI in the evaluation of pulmonary nodules, which consists of a series of fast, non-contrast sequences optimized for nodule detection and characterization [24]. As a one-shot study, MRI may also reduce cost to the healthcare system. Previous studies demonstrate that MRI has a favorable cost effectiveness ratio compared to low-dose CT due to a reduction in false positives and maintaining a similar life expectancy [25]. Our data supports the clinical role of MRI in oncological patients and other promising studies show the potential for MRI as a radiation-free, one-stop analysis for patients with cancer, especially since these patients undergo MR of the abdomen to stage abdominal lesions.

Extrathoracic complications associated with malignancy are a possible finding in oncological patients with pulmonary nodules; this includes adenopathy, cardiomegaly, and pleural effusion [26]. In comparison with CT, our data demonstrates that MRI has an acceptable accuracy in detection of malignant pulmonary nodules as well as these associated complications.

Our study has some limitations. We include 241 participants with 154 nodules being analyzed and only include more common cancers of clinical practice. These limit the power and generalizability of the study, as this could have introduced a selection bias for oncological patients with more severe disease manifestations that inherently require more frequent monitoring. Further, validation of this study’s findings should be performed in more aggressive and non-included cancer etiologies.

## 5. Conclusions

Lung MRI accurately detects pulmonary nodules and other thoracic pathologies commonly observed in oncological patients. Lung MRI shows promise as a valuable tool for oncological patients undergoing routine extrathoracic surveillance, thereby decreasing radiation exposure in especially susceptible populations like pregnant patients and children.

## Figures and Tables

**Table 1:** Patient and nodule characteristics.

Total Patients Patient Age (Years)	241
mean	59.3
median	60 (18–95)
**Extrathoracic neoplasm**	
Breast Cancer	63
Clear Cell Carcinoma	32
Colon Cancer	50
Melanoma	30
Renal Cell Carcinoma	35
Sarcoma	31
**Pulmonary Nodules Detected**	154
Malignant	37
Benign	117
**Nodule Location**	
RUL	40
ML	26
RLL	39
LUL	23
LLL	4
**Average Nodule Size (mm)**	11.5 mm (3.9–29.1)
M1 CT	12.57
M2 CT	10.46
M1 T1	11.79
M2 T1	10.23
M1 T2	12.1
M2 T2	10.49

**Table 2:** Nodule and extrathoracic abnormalities diagnostic value for MRI compared to CT.

	CT	MRI	Sensitivity	Specificity	Accuracy
**Nodules Detected (total)**					
True Positive	154	144	0.9351	1	0.9585
True Negative	87	87			
False Positive	-	0			
False negative	-	10			
**Nodules Detected (>5 mm)**					
True Positive	144	144	1	1	1
True Negative	97	97			
False Positive	-	0			
False negative	-	0			
**Nodule Malignancy (DWI)**					
True Positive	37	37	1	0.9159	0.9375
True Negative	107	98			
False Positive	-	9			
False negative	-	0			
**Adenopathy**					
True Positive	7	4	0.5714	0.9829	0.971
True Negative	234	230			
False Positive	-	4			
False negative	-	3			
**Cardiomegaly**					
True Positive	5	4	0.8	0.9959	0.9917
True Negative	236	235			
False Positive	-	1			
False Negative	-	1			
**Pleural Effusion**					
True Positive	4	4	1	0.9831	0.9834
True Negative	237	233			
False Positive	-	4			
False Negative	-	0			
**Pericardial Effusion**					
True Positive	0	0	-	1	1
True Negative	241	241			
False Positive	-	0			
False Negative	-	0			
**Vertebral Fracture**					
True Positive	8	8	1	0.9959	0.996
True Negative	233	232			
False Positive	-	1			
False Negative	-	0			
**Mean Ao (mm)**	33.6	29.38			
**Mean Pa (mm)**	25.3	21.48			

## Data Availability

The original contributions presented in this study are included in the article. Further inquiries can be directed to the corresponding author.

## Abbreviations

MRI	magnetic resonance imaging
CT	computed tomography
